# Bioinformatics Analysis of Macrophages Exposed to *Porphyromonas gingivalis*: Implications in Acute vs. Chronic Infections

**DOI:** 10.1371/journal.pone.0015613

**Published:** 2010-12-23

**Authors:** Wen-Han Yu, Han Hu, Qingde Zhou, Yu Xia, Salomon Amar

**Affiliations:** 1 Bioinformatics Graduate Program, Boston University, Boston, Massachusetts, United States of America; 2 Center for Anti-inflammatory Therapeutics, School of Dental Medicine, Boston University, Boston, Massachusetts, United States of America; Charité, Campus Benjamin Franklin, Germany

## Abstract

**Background:**

Periodontitis is the most common human infection affecting tooth-supporting structures. It was shown to play a role in aggravating atherosclerosis. To deepen our understanding of the pathogenesis of this disease, we exposed human macrophages to an oral bacteria, *Porphyromonas gingivalis (P. gingivalis*), either as live bacteria or its LPS or fimbria. Microarray data from treated macrophages or control cells were analyzed to define molecular signatures. Changes in genes identified in relevant pathways were validated by RT-PCR.

**Methodology/Principal Findings:**

We focused our analysis on three important groups of genes. Group PG (genes differentially expressed by live bacteria only); Group LFG (genes differentially expressed in response to exposure to LPS and/or FimA); Group CG (core gene set jointly activated by all 3 stimulants). A total of 842 macrophage genes were differentially expressed in at least one of the three conditions compared to naïve cells. Using pathway analysis, we found that group CG activates the initial phagocytosis process and induces genes relevant to immune response, whereas group PG can de-activate the phagocytosis process associated with phagosome-lysosome fusion. LFG mostly affected RIG-I-like receptor signaling pathway.

**Conclusion/Significance:**

In light of the fact that acute infections involve live bacteria while chronic infections involve a combination of live bacteria and their byproducts, group PG could represent acute *P. gingivalis* infection while group LFG could represent chronic *P. gingivalis* infection. Group CG may be associated with core immune pathways, triggered irrespective of the specific stimulants and indispensable to mount an appropriate immune response. Implications in acute vs. chronic infection are discussed.

## Introduction

*Porphyromonas gingivalis* (*P. gingivalis*) is a Gram-negative anaerobic bacterium and is considered to be the major pathogen causing periodontal disease. Periodontitis is one of the most common chronic infectious diseases. It leads to destruction of the attachment apparatus and supporting bone of the teeth. *P. gingivalis* has been shown to invade endothelial cells, followed by activation of pro-inflammatory cells [1]. The bacteria can produce a variety of virulence factors, including lipopolysaccharide (LPS), fimbriae A (FimA), proteinases, outer membrane proteins and specific enzymes [2], [3], which can be involved in initiation and progression of periodontal diseases. *P. gingivalis* LPS is considered to be the primary bacterial component causing the production of pro-inflammatory cytokines and host activation [4], although it shows low endotoxic activity comparing to enterobacterial LPS [5]. *P. gingivalis* FimA is a pivotal factor for mediating the adherence of bacteria to host cell [6]. It mediates the interaction between *P. gingivalis* and Toll-like receptor 2 (TLR2) on the surface of macrophages, and activates another receptor, complement receptor 3 (CR3) for intracellular entry [7], [8]. *P. gingivalis* survival and its ability to cause disease are enhanced in the presence of robust TLR-2/4-dependent host recognition. This phenomenon may explain the persistence of *P. gingivalis* implicated in chronic infectious and inflammatory lesions [9]. In other words, the activation of innate immune signaling pathways by Toll-like receptors may help induce and/or maintain chronic infection [10]. Many studies have been focused on the distinct biological properties of each component in the pathogenesis of periodontal disease, but few have considered the cooperative contributions of coexisting bacteria and virulent components to the development of infection. The present study addressed the influence of individual virulence factors either alone or in combinations, in an effort to explore more complex interactions related to pathogenesis, such as acute and chronic infection.

From three simple macrophage exposure conditions (*P.gingivalis*, LPS and FimA) versus control sample, we propose an *in vitro* model to estimate the combined impact of *P. gingivalis* and/or its virulence factors (i.e. LPS and FimA) on innate immune response in human macrophages, with analysis at the systems level. The macrophage genes which were differentially expressed under each of the conditions versus the control sample were identified. Genes differentially expressed in multiple conditions were visualized in a Venn diagram. In addition, we specified three important groups from the diagram. Functional annotations and pathway analyses of those groups provided valuable insight into unique components of the macrophage response to *P. gingivalis*.

## Results

### Gene comparisons in three microarray conditions

**Figure 1:**
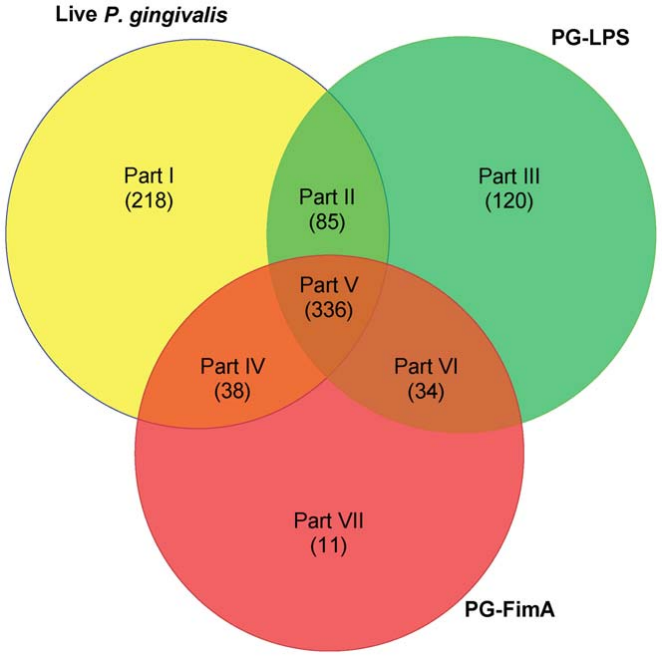
Venn diagram representing the logical relations among the genes from three macrophage treatment conditions. Macrophage genes differentially regulated in response to live *P.gingivalis*, LPS or FimA, compared with controls are shown as yellow, green and red circles, respectively. According to the logical relations, the total gene set was divided into 7 subsets. The number of genes in each part is shown in parentheses. Subset I: Live *P. gingivalis*-only gene set, where genes were only apparent in live *P. gingivalis* treatment. Subset II: The genes were found in both live *P. gingivalis* and LPS treatments, but not in FimA. Subset III: LPS-only gene set. Subset IV: The genes are found in both live *P. gingivalis* and FimA treatment, but not in LPS. Subset V: The genes appeared in three stimuli. Subset VI: The genes found in both LPS and FimA, but not in live *P. gingivalis* treatment. Subset VII: FimA-only gene set.

A total of 842 genes which showed differential expressions compared to the control were identified and collected from the microarrays of three macrophage exposing conditions, including live *P.gingivalis*, LPS and FimA versus control (sterile broth). The genes were identified by the threshold of fold change (FC) and False Discovery Rate (FDR). A Venn diagram representing the distribution of these genes among the three exposure conditions is shown in Figure 1. The regions covered multiple conditions indicate the genes differentially expressed in response to more than one of the exposure conditions. Accordingly, seven subsets of the genes can be identified. Subset I, III and VII show that the sets of the genes specifically respond to a signal stimulus only: live *P. gingivalis*, LPS and FimA, respectively. Subset II, IV and VI indicate the sets of the genes involved in any combination of both two stimuli, suggesting that the genes are shared to react to the stimuli. Subset V presents the set of the genes commonly happen when the macrophages expose to these three stimuli.

Figure 1 shows that macrophages responded to live *P. gingivalis* with the highest number of changes (677) in gene expression, although exposure to individual components also induced significant changes: LPS (575) and FimA (419). There were 336 genes (subset V) shared among all three conditions. Only 11 genes were limited to the FimA-only subset (subset VII) while there were 120 to LPS-only subset (subset III), and 218 to live *P. gingivalis*-only subset (subset I). In addition, 80.2% of the genes for which expression was altered in response to FimA were also differentially expressed in response to both live *P. gingivalis* and to LPS. It has long been suggested that macrophages have evolved with an exquisite sensitivity to the presence of LPS, and activate many response genes specific to activation by LPS.

To delve more deeply into the macrophage response to live *P. gingivalis* versus its LPS and FimA, we focused on three groups for further analysis. The groups of genes were concatenated from the previously described seven subsets in the Venn diagram. *P. gingivalis* group (PG) selected from subset I includes the genes differentially expressed in the *P. gingivalis*-specific condition. The combination of subsets III, VI, and VII was defined as LPS-FimA group (LFG) where the genes are differentially expressed only in the existence of *P.gingivalis* byproducts, which means that the genes triggered by either byproduct were included. Finally, the Core group (CG) extracted from subset V was considered to be a common gene set that is required for general innate immune response. These three groups were used for further pathway analysis.

### Enrichment pathway analysis in group PG, LFG and CG

To gain underlying biological insights from the results of gene expression in these three groups, enrichment pathway analysis was implemented to uncover the major perturbed pathways under the specific groups of differentially expressed genes. There were a total of 842 of genes in three groups; 223 of these genes were functionally clustered in the various categories of KEGG pathways. Other available pathway databases, including PANTHER [11], Reactome [12], and BioCarta, were also implemented for gene annotation clustering. However, KEGG showed the highest coverage among total identified genes. Fisher’s exact test was applied to determine whether the proportion of the genes falling into each category was significant. The list of the mapping pathways is shown in Table 1. *P*-value indicates the probability of forming a group by random chance. We found 13 pathways that matched significantly (*p*<0.001); most of them (11/13) correspond to host defense mechanisms. According to pathway matching, we expected that each of the three stimuli, i.e., live *P.gingivalis*, LPS, fimbriae, would activate the macrophage through TLR2, a member of Toll-like receptor signaling pathway recognizing bacterial pathogen (See Table S1), and also the RIG-I-like receptor signaling pathway, and start to produce and secrete various types of cytokines for inflammatory responses. The secreted cytokines then triggered additional immune responses such as chemokine signaling pathway(s) and Jak-STAT signaling pathway to induce additional responses, e.g. migration, cellular polarization, apoptosis, etc. In the list of the pathways, we found gene sets involved in T cell and B cell receptor signaling pathways, suggesting that the induced macrophage response would be expected to activate T and/or B lymphocytes. The results of mapping pathways illustrated that live *P.gingivalis* and its cell components were able to initiate innate and adaptive immune responses.

**Table 1:** The list of pathway category mapping from three groups of genes.

Pathway ID	Pathway name	Count^1^	Percent^2^	P-Value^3^	Fold enrichment	Fisher exact	CG 4	PG^4^	LFG^4^
04620	Toll-like receptor signaling pathway	22	3.1	1.30E-09	5	2.10E-10	54.5%	22.7%	22.7%
04060	Cytokine-cytokine receptor interaction	35	4.9	5.20E-09	3	1.60E-09	80.0%	5.7%	14.2%
04621	NOD-like receptor signaling pathway	16	2.2	3.70E-08	5.9	5.00E-09	93.7%	6.3%	0.0%
04622	RIG-I-like receptor signaling pathway	14	1.9	9.00E-06	4.5	1.70E-06	50.0%	7.1%	42.8%
04623	Cytosolic DNA-sensing pathway	12	1.7	1.90E-05	5	3.10E-06	66.6%	8.3%	25.0%
04210	Apoptosis	15	2.1	1.90E-05	3.9	4.20E-06	60.0%	26.6%	13.3%
04630	Jak-STAT signaling pathway	19	2.6	1.20E-04	2.8	3.90E-05	57.8%	21.1%	21.1%
04062	Chemokine signaling pathway	20	2.8	4.50E-04	2.4	1.70E-04	65.0%	30.0%	5.0%
05221	Acute myeloid leukemia	10	1.4	8.00E-04	3.9	1.80E-04	50.0%	40.0%	10.0%
05200	Pathways in cancer	27	3.8	2.00E-03	1.9	9.90E-04	59.2%	25.9%	14.8%
04010	MAPK signaling pathway	23	3.2	2.70E-03	2	1.30E-03	69.5%	21.7%	8.6%
04662	B cell receptor signaling pathway	10	1.4	5.00E-03	3	1.50E-03	60.0%	30.0%	0.0%
04660	T cell receptor signaling pathway	12	1.7	7.10E-03	2.5	2.50E-03	58.3%	41.6%	0.0%

1 Total count of genes from three groups.

2 The proportion of total count versus the number of genes in the pathway.

3 Only the mapped pathways with *p*-value <0.001 were shown.

4 PG indicates *P.gingivalis*-specific group. LFG indicates LPS and FimA-specific group, and CG presents core group sharing in *P.gingivalis* and it byproducts. The relative proportion of each group in the corresponding pathway category.

Notably in the NOD-like receptor signaling pathway responsible for detecting pathogens and generating the innate immune response, 93.7% of identified genes were shared (from CG); only 6.3% were found in PG and not in LFG, suggesting that this fundamental pathway can be triggered by different conditions of infection. For the RIG-I-like receptor signaling pathway, PG (7.1%) was poorly represented whereas 42.8% of genes in the pathway came from LFG, suggesting that several key genes involved in the pathway are only activated by *P.gingivalis* byproducts.

### Gene regulation in three groups confirmed by quantitative real-time PCR

**Figure 2:**
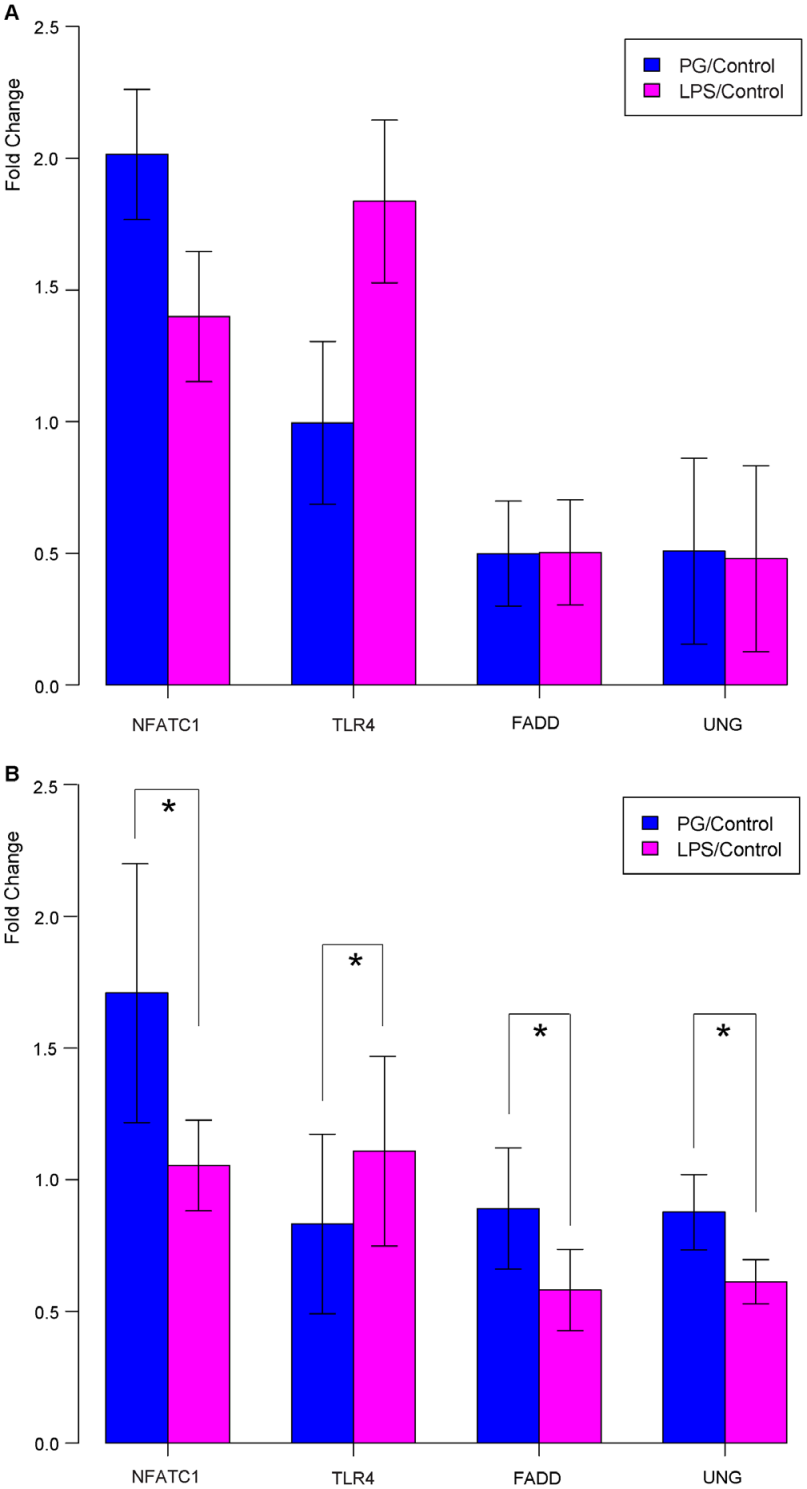
Real-time PCR demonstrates that relative transcription levels for *NFATC1*, *UNG*, *FADD* decrease in PG/LPS treatment while the level for *TLR4* increases. **A.** Comparison of gene expression between PG/Control and LPS/Control from microarray study. The *FADD* gene and *UNG* gene were selected for validation due to important host gene responding to *P.gingivalis* infection. **B.** Comparison of gene expression between PG/Control and LPS/Control from real-time PCR. Asterisks indicate statistically significant (p<0.05) differences in change of mRNA expression. The fold change is log2 scale.

From those significantly regulated pathways identified by enrichment analysis, several key regulated genes that only mapped to either PG or LFG were selected and further validated by quantitative real-time PCR (Figure 2, Table 2). In order to quantitatively catch the relative changes of differential gene expressions between PG/control and LPS/control, we parallelly included FC ratio from both qPCR and microarray in Table 2 in which PG/control divided by LPS/control. The *NFATC1* gene, selected from PG of the three groups, functioned as a transcription factor for cytokine genes to activate T cell immune response and showed up-regulation in both PG/control and LPS/control from microarray data (Figure 2A). However, PG/control had higher degree of fold change compared to LPS/control which was confirmed by qPCR results (Figure 2B). TLR4, which sensitively detects LPS on Gram-negative bacteria and activates the innate immune system, was identified from LFG, and the gene was confirmed by qPCR that highly expression in LPS/control compared to PG/control. The *CD40* gene, functioning as a inflammatory cytokine induced by TLR2/TLR4- dependent Toll-like receptor signaling pathway [13], exhibited higher up-regulation in PG/control than in LPS/control consistent with the fact that *P. gingivalis* is known to predominantly engage TLR2 as well as TLR4. However, in qPCR result, the value of up-regulation in PG/control was less than in LPS/control. FADD is an adaptor molecule transmitting signals for apoptosis which may also be important for early T cell development; CASP6 functions as apoptosis-related cysteine peptidase. Both genes showed higher up-regulation in PG/control from qPCR data. The *SOCS1* gene, functioning as a key physiological component directly interacting with cytokine regulators to the immune system, displayed higher up-regulation in LPS/control. In addition, PKC (protein kinase C), PI3K (phosphoinositide-3-kinase), and PKA (protein kinase A) found in multiple pathways, including chemokine signaling, apoptosis, Jak-STAT signaling, and B cell and T cell receptor signaling. Both *PKC* and *PKA* genes showed higher up-regulation in PG/control, whereas *PI3K* gene expressed higher up-regulation in LPS/control in both qPCR and microarray data. ArfGEF plays an important role in vesicular transport for endocytosis, and UNG, uracil-DNA glycosylase, located in the pathway of primary immunodeficiency, and both genes showed higher up-regulation in PG/control confirmed by qPCR.

**Table 2:** qPCR validation for selected genes from PG and LFG.

Gene symbol	P-value	FC ratio^1^ by qPCR	FC ratio^1^ by microarray	Group	EntrezID	Gene name	Pathway ID^2^
*NFAT*(*NFATC1*)	0.0250	1.58	1.43	PG	4772	Nuclear factor of activated T-cells, cytoplasmic, calcineurin-dependent 1	04660; 04662
*TLR4*	0.1771	0.72	0.54	LFG	7099	Toll-like receptor 4	04620
*CD40*	0.7309	0.82	1.17	PG	958	CD40 molecule, TNF receptor superfamily member 5	04060; 04620
*FADD*	0.0419	1.54	0.99	LFG	8772	Fas (TNFRSF6)-associated via death domain	04210; 05200
*CASP6*	0.7603	1.25	1.03	PG	839	Caspase 6, apoptosis-related cysteine peptidase	04210
*SOCS1*	0.2653	0.66	0.33	LFG	8651	Suppressor of cytokine signaling	04630
*PKC*(*PRKCD*)	0.6690	1.22	1.52	PG	5580	Protein kinase C, delta	04062;
*PI3K*(*PI3KR5*)	0.2193	0.92	0.50	PG	23533	Phosphoinositide-3-kinase, regulatory subunit 5	04210; 04620; 04630; 04660; 04662; 05200; 05221
*PKA*(*PRKACB*)	0.7916	1.42	1.41	PG	5567	Protein kinase, cAMP-dependent, catalytic, beta	04010; 04062; 04210
*ArfGEF* (*PSD5*)	0.3661	1.13	1.22	PG	23362	Pleckstrin and Sec7 domain containing 3	04144
*UNG*	0.0098	1.43	1.06	LFG	7374	Uracil-DNA glycosylase	05340

1 FC ratio here indicated the ratio of PG/control over LPS/control.

2 The genes involved in the pathways showed in the Table1.

## Discussion

The present paper provides a comprehensive analysis of gene expression from microarray results of macrophages when exposed to live *P. gingivalis* and compared to its byproducts: LPS or fimbriae. Our bioinformatics approach allowed the identification of gene sets solely expressed when live bacteria are exposed to macrophages as opposed to bacterial by-products alone or in combination. Three groups of differentially regulated genes were identified: PG, LFG, and CG. The PG group was selected from subset I, with genes differentially expressed only in response to exposure to live *P. gingivalis*-specific condition. Group LFG, the union of subsets III, VI, and VII, included the genes whose regulation appeared to be altered only in the response to *P.gingivalis* byproducts. Group CG represents the gene set activated in both conditions, which we suggest forms the backbone of human macrophage immune responses to *P.gingivalis* infection. Further study of these groups may identify key genes involved in acute and chronic stages during infection as well as the shared mechanisms in both conditions. Our main observation proposes novel clues potentially involved in phagocytosis during infection process.

We then analyzed the individual contribution of each group among the mapped pathways (Table 1). CG certainly contributed the greatest proportion (>50%) to each pathway, referred to here as the core genes responding to general infection. Also, this result suggests that the host maintains a conserved defense mechanism core against different types of bacterial stimuli.

### Specific pathways induced by Group PG

A key transcription factor – NFAT, which controls the production of cytokines for cell proliferation, differentiation and immune response, displayed up-regulation only in response to live *P.gingivalis* infection. NFAT could thus be an early signal for development of adaptive immune response after live *P.gingivalis* infection. In addition, several genes up-regulated in CG: *PKC*, *MARCKS*, and *PAP*, (See Table S1) relate to the activation of phagocytosis associated with endocytosis, whereas down-regulation of *Cofilin* in PG (See Table S1) might inhibit the phagocytosis process associated with phagosome-lysosome fusion. Combination of each piece of information suggested that this phenomenon is similar to the pathogen intracellular invasion [14]. Those pathogens can enter macrophages through lipid rafts, cholesterol-enriched microdomains for cellular trafficking and signaling transduction, and prevent the fusion of phagosome with late endosome/lysosome [15]. Recently, Wang *et al*. found that *P. gingivalis* can also invade macrophage via lipid rafts [16]. The present data provide novel clues regarding the molecular control mechanism in phagocytosis pathway during the infection process.

### Specific pathways induced by Group LFG

It is notable that in the RIG-I-like receptor signaling pathway 42.8% of genes were found in LFG but only 6.3% in PG. A small cluster of genes (*RIG-I*, *MDA5*, *LGP2* and *ISG15*; See Table S1) from LFG was mapped in this pathway, which is responsible for recognizing viral RNA and activating NF-κB, MAP kinases, and IRFs that control the release of inflammatory cytokines [17]. Recently, Kong *et al.* found that LPS from *E. coli*, by binding to TLR4, was able to induce the expression of RIG-I in macrophages which then activated LPS-induced phagocytosis [18]. Wang *et al.* found that the LPS-stimulated expression of TNF-α in macrophages can be divided into an early phase and a late phase, wherein the late phase is promoted by induction of RIG-I [19] while our experimental data included macrophage exposure to trigger for 2 hours reflecting early phase and future studies will include later time-points to reflect late phases. Until now, it is not clear if there is any correlation between RIG-I induced phagocytosis and the special “lipid raft” style of internalization of *P. gingivalis,* which could finally lead to other systematic chronic diseases. These results suggest an assistant role played by LPS when *P. gingivalis* invades the macrophage cells; in another words, LPS could help initiate and maintain the phagocytosis process.

### General inflammatory pathways shared in Group CG

**Figure 3:**
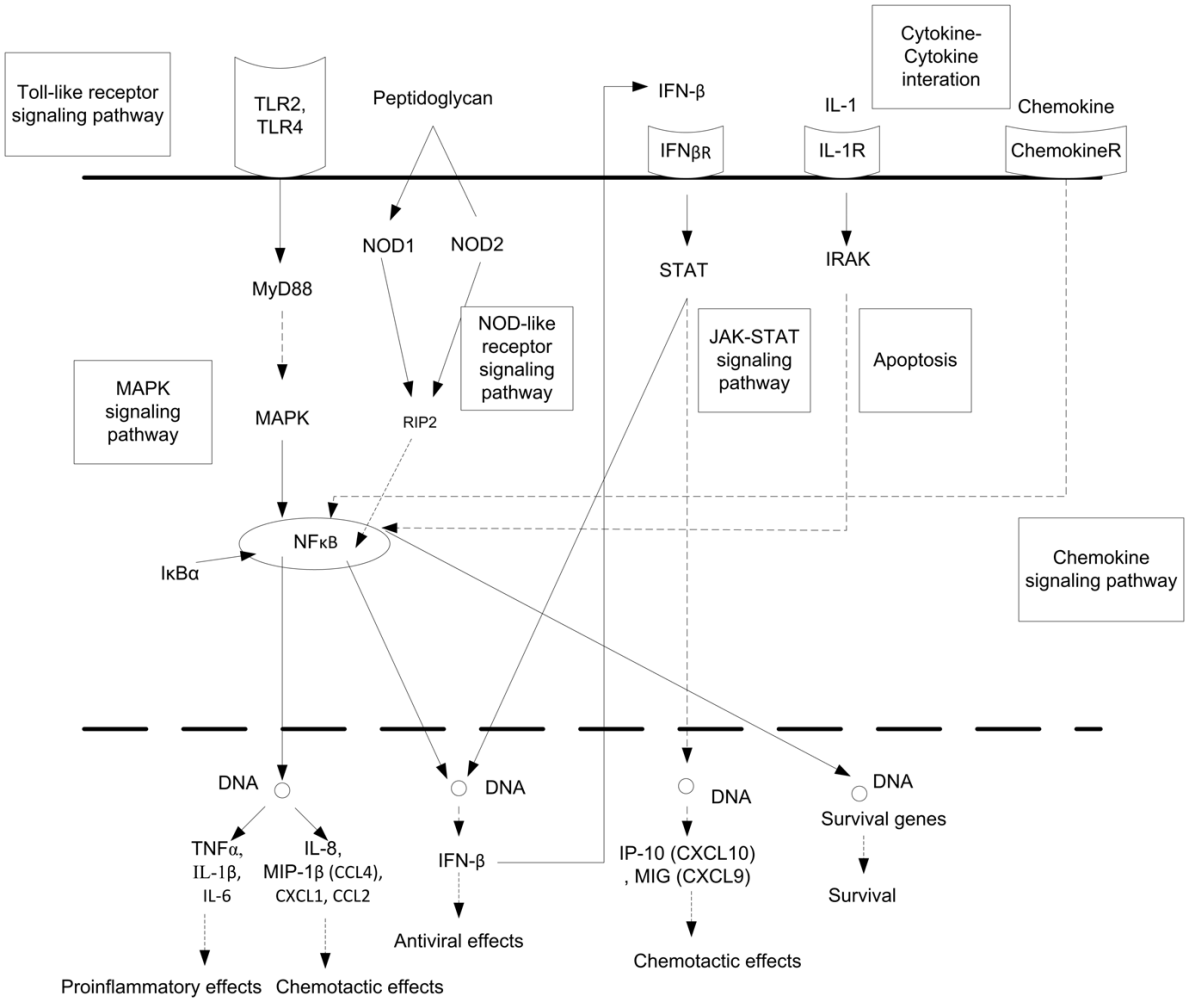
The overall framework of the innate immune response triggered by all three stimuli. Pathways in CG were used in constructing the general regulatory pathway. Not all the genes shown in this pathway were up- or down-regulated across three conditions. 7 pathways were involved: Toll-like receptor signaling pathway, NOD-like receptor signaling pathway, Jak-STAT signaling pathway, MAPK signaling pathway, apoptosis, chemokine signaling pathway, and cytokine-cytokine receptor interaction.

From pathway analysis, CG represents the general signaling pathways for innate immune response induced by exposure to these three stimuli. *P. gingivalis* and its virulent byproducts bind to TLRs (mostly TLR2) and induce NFκB-dependent gene expression via the MyD88-p38 MAPK pathway. The activation of NFκB and MAPK is quick and helps to produce pro-inflammatory cytokines, such as TNFα, IL-1β and IL-6. The NOD-like receptor (NLR) family plays an important role in intracellular ligand recognition, followed by activation of NFκB and MAPK, cytokine production and apoptosis pathways. The produced cytokines can trigger innate or adaptive immune responses by binding to specific receptors on macrophage and other immune cell surfaces and resulting in host defense, cell growth, differentiation, and cell death. The main pathways involved are summarized in Figure 3, which includes Toll-like receptor, MAPK, NOD-like receptor, Jak-STAT, chemokines and apoptosis signaling pathways, and cytokine-cytokine receptor interactions.

### Biological insight from the differential pathways

Phagocytosis has been known to be an important process in host defense. In addition, for pathogenic invasion it is also an initial stage of local chronic disease that sometimes develops into distal chronic disease [8], [20], [21]. The interaction between pathogen and host cells can be divided into three situations: 1) the pathogen camouflages itself to prevent phagocytosis (local chronic infection); 2) the macrophage captures the pathogen by phagocytosis, and the phagosome successfully fuses with lysosome (acute infection that resolves); 3) the pathogen is phagocytosed but prevents the fusion between phagosome and late endosome/lysosome (local and distal chronic infection). Several studies have revealed the critical role played by FimA in the internalization of *P. gingivalis* into macrophages [7], [22]. In our study, *RIG-I* and its neighbor genes in the RIG-I-like receptor signaling pathway were significantly up-regulated in macrophages exposed to bacterial components but not live *P. gingivalis* (the LFG but not the PG). This result suggests that live *P. gingivalis*- or its byproducts may induce phagocytosis via distinct mechanisms, *e.g.* RIG-I-independent without fusion, vs. RIG-I-dependent with phagosome-lysosome fusion, which might pertain to the mechanisms distinguishing between acute and chronic infection.

### Implications in Acute vs. Chronic infections

Given the facts that administration of live bacteria through multiple routes replicates many of the characteristics seen in acute infections such as clinical sepsis [23] while chronic infections being a local insult are usually obtained in animals using bacteria products such as LPS or fimbria or very low concentration of live bacteria. [23], [24], [25] we speculate that the gene set representing that PG could reflect genes expressed acute infection while LFG could reflect genes expressed in more chronic infections. Indeed septic shock which is the epitome of acute infection in animals is mostly obtained after administration of live bacteria; in contrast the use of LPS to generate an endotoxic response requires the animals to be artificially pre-sensitized using drugs like D-galactosamine which inflict liver toxicity and does not mimic clinical settings [26], [27].

By studying the host responses stimulated by exposing human macrophages to either live *P. gingivalis*, LPS and FimA, we identified pathways involved in both general innate immune response, as well as potentially distinct effects on phagocytosis induced by different stimuli. This approach may help to discern the connection between infectious environments and infection phase transition from acute to chronic stage. Identification of the molecular signature from PG, LFG and CG may one day help us distinguish between acute and chronic infection in clinical diagnosis.

## Materials and Methods

### Bacterial strain and macrophage preparation

*P. gingivalis* 381 (ATCC) was cultured anaerobically, as described previously [28]. Protein-free LPS from *P*. *gingivalis* 381 was extracted with phenol-water and purified by cesium chloride isopyknic density gradient ultracentrifugation followed by re-purification, and FimA was purified by size exclusion chromatography, as previously described [28]. The preparation of macrophage culture is described in Zhou et al [29].

### Exposure of macrophages with *P. gingivalis* and its components

Adherent macrophages were infected with live *P*. *gingivalis* at indicated multiplicities of infection (MOI). Live *P*. *gingivalis* 381 were diluted in media to a concentration of 5×10^8^ bacteria per 50 µl, and added to cultures of macrophages. Purified LPS or FimA from *P. gingivalis* was added to cell culture media at indicated concentrations. Cells were incubated at 37°C in an atmosphere containing 5% CO_2_. Cells and supernatants were harvested for 2 hours after incubation with live *P. gingivalis* or its components.

### RNA preparation and microarray analysis

Two hours after infection with *P. gingivalis*, or treatment with *P. gingivalis* LPS, FimA, or saline (control), human macrophages were washed 3 times with ice cold PBS, and total RNA was extracted using an RNeasy Mini Kit according to the manufacturer’s instructions. Three independent experiments were performed for each condition. Synthesis of cDNA first and second strand was performed using the GeneChip Expression 3’-Amplification Reagents One-Cycle cDNA Synthesis Kit (P/N 900431). *In vitro* transcription (IVT) was performed using the GeneChip Expression Amplification Reagents kit-30 reactions (P/N 900449) and was carried out according to the standard Affymetrix protocols. Twenty micrograms of IVT material were used on each GeneChip Human Genome U133 Plus 2.0 array, which contains approximately 54674 gene probe sets. Hybridization and wash steps were based on the Affymetrix GeneChip Manual. Scanning was performed by the GeneChip Scanner 3000 7G scanner with Affymetrix GCOS v1.3 operating system.

### Data processing

The raw Affymetrix CEL files from three replicates for each condition were collected. The Bioconductor (open source software framework) packages running under R platform (a free statistical software environment) were used to process the raw data [30]. First the raw data were read from the CEL files into the object of class AffyBatch. We compared the expression of genes in macrophages under three conditions: live *P*. *gingivalis-*stimulated versus control, LPS-stimulated versus control, and FimA-stimulated versus control. For each condition, background correction and quantile normalization were adjusted by Robust Multichip Average (RMA) in the *Affy* package [31], followed by non-specific filtering (nsFilter) to eliminate the uninformative probe sets whose inconsistent phenotypes appeared in the three replicated arrays. The calculation of fold change and adjusted *p*-value (false discovery rate, FDR) under different conditions was implemented by *limma* package [32]. We selected differentially expressed genes, both up and down regulation, with fold change ≥2 and FDR <0.25 for further analysis. The normalized microarray data and the gene lists among the subsets can be accessed in supplementary materials. The microarray data has submitted to Gene Expression Omnibus database (GEO) and can be public access by GEO accession number- GSE24897.

### Pathway analysis

The three gene groups (PG, LFG and CG) were imported into DAVID [33] for pathway enrichment analysis. The purpose of pathway enrichment analysis is to identify pathways with a significant number of differentially expressed genes, and thereby connect those genes with corresponding functional feature, *i.e*. a pathway. Moreover, the information of up- and down-regulation of those genes as well as their positions in the pathways can provide indication of the positive or negative outcome of the pathway [34], [35], which is instrumental to estimate the impact exerted by the differentially expressed genes on the pathway. Fold enrichment (FE) is used to estimate the enrichment degree of a given pathway, which is defined as
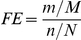
where *m* denotes the number of “hits”(mapped genes) in the pathway, *M* denotes the number of genes in the pathway, *n* denotes the number of “hits” in the background and *N* denotes the number of total genes in the background. The p-values are estimated based on Fisher exact test and its modified version—EASE score [36], respectively.

DAVID [33] is an online database providing services for functional annotation and function classification. In the functional annotation section of DAVID, KEGG pathway (an online tool provided by Kyoto Encyclopedia of Genes and Genomes [37]) was selected as the only option for the enrichment analysis with the background gene set as Human Genome U133 Plus 2.0 Array. DAVID generated a list of enriched KEGG pathways for each uploaded gene set, as well as the corresponding information of *p*-value, count, percentage (%), and fold enrichment for each KEGG pathway. The *p*-value here stands for EASE score, a modified Fisher exact p-value, which reflects the probability that the uploaded list is associated with specific KEGG pathway by random chance. The pathways with *p*-value <0.001 were considered as significantly enriched pathways. We selected the pathways from the enrichment list and focused on the differentially expressed genes which distributed at the neighborhood on the pathway topology. We also provided a color scheme to present either up- or down-regulation of expression of the genes that mapped to KEGG pathways by KEGG IDs converted from Entrez gene IDs of those genes. A Perl script was used to generate pathway image files with colored genes through KEGG API.

### Quantitative Real Time-PCR validation

We selected 12 of genes mapped to different KEGG pathways for our microarray validation. The RNA was extracted from human macrophages with QuickGene RNA tissue kit SII and QuickGene-810, after macrophage 2-hour infection with *P. gingivalis* strain 381, treatment with LPS or saline as described before. Reverse transcription of RNA (1.5 µg) in each 30 µl of reaction was performed using iScript cDNA Synthesis kit (Bio-Rad) according to the manufacturer’s protocol. Primer sequences specific to 12 of target genes were generated with Beacon Designer software (Bio-Rad). Quantitative PCR was conducted using the iQ SYBR Green Supermix (Bio-Rad) in Bio-Rad iCycler. The amounts of mRNA expression from three independent samples were normalized to that of GAPDH (Glyceraldehyde 3-phosphate dehydrogenase).

## Supporting Information

Table S1 **The microarray gene expression data from three conditions.** The list of gene expression in three conditions shows six types of fold changes, including *P. gingivalis*/Control, LPS/Control, FimA/Control, *P. gingivalis*/LPS, *P. gingivalis*/FimA and LPS/FimA.
(XLS)
